# A qualitative analysis of family caregiver perspectives from the Caregiving Transitions Study

**DOI:** 10.1093/geroni/igab046.2917

**Published:** 2021-12-17

**Authors:** Marcela Blinka, Chelsea Liu, Orla Sheehan, J David Rhodes, David Roth

**Affiliations:** 1 Johns Hopkins University, Baltimore, Maryland, United States; 2 Harvard School of Public Health, Allston, Massachusetts, United States; 3 Johns Hopkins University School of Medicine, Johns Hopkins University, Maryland, United States; 4 University of Alabama at Birmingham, University of Alabama at Birmingham, Alabama, United States

## Abstract

As people live longer, informal caregiving for family and friends is becoming increasingly common. Caregiver satisfaction with their role is now of greater importance to an increasing proportion of the U.S. population. Most research on caregivers has studied convenience samples, often restricted to caregivers of people with dementia. Various studies have examined the impact of caregiving on caregivers’ health but to our knowledge there are no qualitative studies of caregiving experiences from caregivers in population-based samples. This study investigated the impact of caregiving on participants who transitioned into a caregiving role while participating in a national population-based study. Participants were from the Caregiving Transitions Study, which is ancillary to the Reasons for Geographic and Racial Differences in Stroke (REGARDS) Study. We thematically analyzed responses from 150 caregivers providing care for multiple different conditions to an open-ended question asked at the time of enrollment and designed to encourage caregivers to share additional details about their caregiving experience. Four major themes were identified: cultural/family expectations; growth opportunities and reciprocity; stressors and challenges; and recommendations. Participants shared both positive and challenging experiences in their role as a family caregiver as well as the impact that these experiences had on their lives. Caregivers shared that one of the most important motivations for taking on this role was their sense of duty toward family. Caregivers also highlighted the positive impact of caregiving on their lives such as opportunities for personal growth, acquisition of new skills, and finding a sense of fulfillment and gratitude.

